# Free Mg^2+ ^concentration in the calf muscle of glycogen phosphorylase and phosphofructokinase deficiency patients assessed in different metabolic conditions by ^31^P MRS

**DOI:** 10.1186/1476-5918-4-7

**Published:** 2005-06-06

**Authors:** Emil Malucelli, Raffaele Lodi, Andrea Martinuzzi, Caterina Tonon, Bruno Barbiroli, Stefano Iotti

**Affiliations:** 1 Dipartimento di Medicina Clinica e Biotecnologia Applicata, Università di Bologna; Via Massarenti 9, 40138 Bologna, Italy

## Abstract

**Background:**

The increase in cytosolic free Mg^2+ ^occurring during exercise and initial recovery in human skeletal muscle is matched by a decrease in cytosolic pH as shown by *in vivo *phosphorus magnetic resonance spectroscopy (^31^P MRS). To investigate *in vivo *to what extent the homeostasis of intracellular free Mg^2+ ^is linked to pH in human skeletal muscle, we studied patients with metabolic myopathies due to different disorders of glycogen metabolism that share a lack of intracellular acidification during muscle exercise.

**Methods:**

We assessed by ^31^P MRS the cytosolic pH and free magnesium concentration ([Mg^2+^]) in calf muscle during exercise and post-exercise recovery in two patients with McArdle's disease with muscle glycogen phosphorylase deficiency (McArdle), and two brothers both affected by Tarui's disease with muscle phosphofructokinase deficiency (PFK).

**Results:**

All patients displayed a lack of intracellular acidosis during muscle exercise. At rest only one PFK patient showed a [Mg^2+^] higher than the value found in control subjects. During exercise and recovery the McArdle patients did not show any significant change in free [Mg^2+^], while both PFK patients showed decreased free [Mg^2+^] and a remarkable accumulation of phosphomonoesters (PME). During initial recovery both McArdle patients showed a small increase in free [Mg^2+^] while in PFK patients the pattern of free [Mg^2+^] was related to the rate of PME recovery.

**Conclusion:**

i) homeostasis of free [Mg^2+^] in human skeletal muscle is strongly linked to pH as shown by patients' [Mg^2+^] pattern during exercise;

ii) the pattern of [Mg^2+^] during exercise and post-exercise recovery in both PFK patients suggests that [Mg^2+^] is influenced by the accumulation of the phosphorylated monosaccharide intermediates of glycogenolysis, as shown by the increased PME peak signal.

iii) ^31^P MRS is a suitable tool for the *in vivo *assessment of free cytosolic [Mg^2+^] in human skeletal muscle in different metabolic conditions;

## Background

Human skeletal muscles contain approximately 35% of total human body magnesium, which is an essential cofactor in a number of cell reactions. Magnesium ions influence the equilibria of many reactions involved in cellular bioenergetics by interacting with phosphorylated molecules and interfere with the kinetics of ion transport across plasma membranes [1]. There is considerable evidence that Mg^2+ ^is actively transported and regulated, although the mechanisms are still largely unknown [2]. In skeletal muscle variations of cytosolic pH, phosphocreatine (PCr) and inorganic phosphate (Pi) concentrations influence the complex multi-equilibrium system of the molecular species binding magnesium ions. As a consequence [Mg^2+^] changes considerably in different metabolic conditions such as rest, exercise and recovery, showing an increase matched by a decrease of intracellular pH during exercise and recovery [3].

We assessed the cytosolic pH and the [Mg^2+^] by ^31^P MRS at rest, during exercise and post-exercise recovery in the calf muscle of two patients with McArdle's disease with muscle glycogen phosphorylase deficiency (McArdle), and two brothers affected by Tarui's disease with muscle phosphofructokinase deficiency (PFK).

These two type of glycogenosis, being characterized by almost absent activity of enzymes involved in glycogenolysis (McArdle) and glycolysis (PFK) pathways, show in general limited/absent production of intracellular lactic acid, depending on the degree of enzyme deficit [4,5]. As consequence, patients with McArdle's and Tarui's disease, typically show a decrease or a lack of intracellular acidification during muscle exercise when studied by ^31^P MRS [6,7]. We used these diseases as natural experimental models to study the pattern of free Mg^2+ ^during exercise and recovery in the absence of intracellular acidification to understand to what extent homeostasis of intracellular free Mg^2+ ^is linked to pH.

## Methods

### Patients

We studied 4 patients: two unrelated males both aged 42, with myo-phosphorylase deficiency (named MCArdle I and II respectively) and two brothers aged 18 and 10 years with phosphofructokinase deficiency (named PFK I and II respectively), as detected by histochemical/biochemical analysis of muscle.

Ten healthy volunteers (10 males age: 33 ± 15) were recruited as control subjects. Written informed consent was obtained from all subjects.

### Protocol

MR spectra were acquired on a General Electric 1.5 T Signa System whole-body scanner. Radiofrequency pulses at 25.866 MHz with a pulse width of 400 μs and a transmitter power of 0.5 kW were transmitted by a surface coil (20.5 cm diameter; General Electrics) and the resonance signals were collected by a 7.5 cm receiving coil. A data table of 1024 complex points was collected for each FID. The band width was 2 kHz. The delay between transmission and reception was 0.5 ms and the dwell time was 250 μs. The stimulation-response sequence was repeated every 5000 ms (TR = 5000 ms). Magnetic field homogeneity was optimized by shimming the ^1^H water spectrum (FWMH 0.25–0.35 ppm)

The spectroscopic measurements were performed according to the quantification and quality assessment protocols defined by the EEC Concerted Research Project on "Tissue Characterisation by MRS and MRI", COMAC-BME II.1.3 [8].

Subjects lay supine with a 20.5/7.5 cm diameter transmitter/receiver surface coil centred on the maximal circumference of the right calf muscle. Muscle aerobic incremental exercise consisted of different levels of 1 minute each (12-FIDs) of plantar flexion against a pedal using a pneumatic ergometer [9]. All patients were asked to perform an exercise to reach a PCr depletion of about 50% at the end of exercise. Sixty-four FIDs at rest, and 12 FIDs for each level of work were averaged. During recovery 4-FIDs data blocks (20 s) were recorded for 60 s, while longer time blocks were collected thereafter. The area of each metabolite signal was fitted to a Lorentzian line shape using a time-domain fitting program AMARES/JMRUI[10], the PCr and Pi concentration were calculated by assuming a normal ATP concentration of 8 mM [11]. The cytosolic pH and [Mg^2+^] are calculated from the chemical shift of Pi and β-ATP respectively, both measured from the resonance of PCr, using an equation which takes into account the mutual influence between pH and [Mg^2+^] [3]. The simultaneous calculation of [Mg^2+^] and pH was performed by the specific software package MagicMC, that we developed and made available on the internet [12].

## Results

^31^P MRS spectra of human skeletal muscle typically show the peak signal of: phosphomonoesters (PME) which represents the phosphorylated monosaccharide intermediates of glycogenolysis, inorganic phosphate (Pi), phosphocreatine (PCr) and the three phosphate groups α, β, γ of ATP.

**Figure 1:**
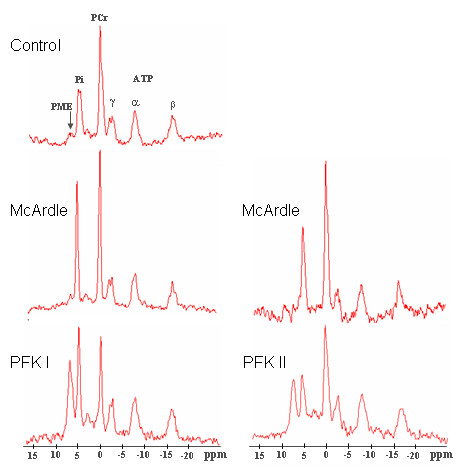
**^31^P MRS spectra of calf muscle during exercise. **End-exercise ^31^P MRS spectra of calf muscle acquired in patients and in a control subject reaching a PCr depletion of about 50%. PFK patients showed a marked phosphomonoester (PME) accumulation, although to a different extent.

Figure 1 shows ^31^P MRS spectra acquired at the end of exercise in the calf muscle of McArdle and PFK patients compared to that of a control subject reaching a similar level of PCr depletion. End exercise spectra of both PFK patients show a marked increase in the PME peak.

Table 1 reports the rest and end-exercise values of cytosolic [Mg^2+^] and pH in patients' calf muscle, compared to the mean values obtained in a control group of ten subjects with a comparable end-exercise PCr depletion. At rest all patients showed a cytosolic pH not different from control values. Resting cytosolic [Mg^2+^] in PFK II patient was 0.45 mM, higher than the control value (0.32 + 0.04 mM) [3], while in PFK I and both McArdle's patients was normal. Both McArdle and PFK I patients reached a similar PCr depletion just above 50%, while PFK II patient stopped at a lower degree of PCr depletion. All patients displayed a lack of intracellular acidosis during exercise, showing pH values higher than controls at the end of exercise. Cytosolic free [Mg^2+^] at the end of exercise was lower in both PFK patients compared to control values. The variation of free [Mg^2+^] from rest to end-exercise (Δ[Mg^2+^]) was negative in both PFK patients and in McArdle II patient.

**Table 1:** [Mg^2+^] and pH values at rest and end-exercise

	Rest		End-Exercise			
	[Mg^2+^](mM)	pH	%PCr	[Mg^2+^](mM)	pH	Δ[Mg^2+^] (mM)
McArdle I	0.34	6.98	53.1%	0.39	7.06*	+ 0.05
McArdle II	0.36	6.95	53.8%	0.32	7.07*	- 0.04*
PFK I	0.38	6.95	54.1%	0.23*	7.01*	- 0.15*
PFK II	0.45*	6.95	35.3%	0.23*	7.01*	- 0.22*
Controls Mean (*n = 10*)	0.31	6.96	47.7%	0.42	6.8	+0.11
S.D.	0.04	0.02	13.8%	0.06	0.09	0.07
control range [min:max]	[0.27:0.40]	[6.94:7.00]	[22:73]	[0.35:0.54]	[6.90:6.67]	[+0.03:+0.24]

Rest and end-exercise values of cytosolic [Mg^2+^] and pH assessed by ^31^P MRS in patients calf muscle, compared to the mean values obtained in a control group of ten healthy subjects with a comparable end-exercise PCr depletion;

Δ[Mg^2+^]: difference between end-exercise [Mg^2+^] and rest [Mg^2+^];

%PCr: percentage of PCr depletion

* denotes values out of the range: control mean ± 2 SD.

**Figure 2:**
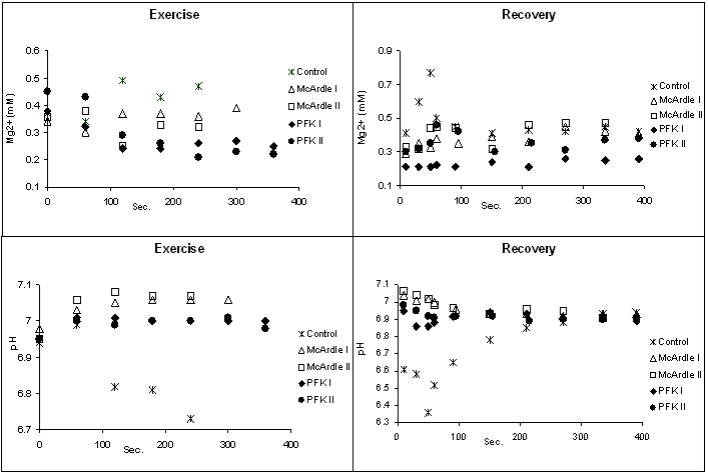
**[Mg^2+^] and pH patterns during exercise and recovery. **Patterns of cytosolic free magnesium concentration and pH at rest, during exercise and recovery in patients compared with typical patterns from a healthy volunteer with comparable PCr depletion. (A): pattern of [Mg^2+^] during exercise; (C): pattern of [Mg^2+^] during recovery; (B): pH pattern during exercise; (D): pH pattern during recovery.

Figure 2 reports the patterns of cytosolic free [Mg^2+^] and pH obtained in patients during exercise (panel A and B) and recovery (panel C and D) compared with typical patterns from a healthy volunteer with comparable PCr depletion. During exercise and recovery the McArdle patients did not show any significant change (McArdle II) or small change (McArdle I) in free [Mg^2+^], while both PFK patients showed decreased free [Mg^2+^] during exercise. On the other hand, during recovery the pattern of free [Mg^2+^] was different in the two PFK patients, with PFK II showing a moderate increase during early recovery.

**Figure 3:**
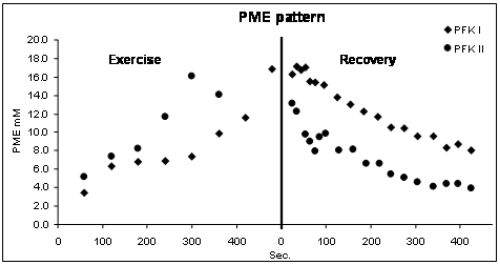
**PME patterns of PFK patients during exercise and recovery. **PFK I patient shows both a slower rate of PME accumulation during exercise and a slower recovery of PME after exercise compared to PFK II patient. PME signal comes from the phosphorylated monosaccharide intermediates of glycogenolysis.

Figure 3 report the PME pattern of the two PFK patients during exercise and recovery. PFK I patient shows a slower rate of both PME accumulation and recovery compared to PFK II patient.

## Discussion

In the skeletal muscle variations of cytosolic pH, phosphocreatine and inorganic phosphate concentrations influence the complex multi-equilibrium system of the molecular species which bind magnesium ions. As a consequence free cytosolic [Mg^2+^] can change considerably in different metabolic conditions such as rest, exercise and recovery. It has been shown by ^31^P MRS that the increase of cytosolic free [Mg^2+^] occurring in skeletal muscle of healthy subjects during exercise and initial recovery is matched by a decrease in cytosolic pH, and the changes in cytosolic free [Mg^2+^] were mainly the result of the predominant effect of [H^+^]. [3]. This result was attributed to mechanisms of binding competition existing between Mg^2+ ^and H^+ ^towards the molecules negatively charged present in the cell cytosol [3]. However, the causal relationship between pH and [Mg^2+^] has not been proved yet, as it could be argued that muscular exercise *per se *elicits an increase in cytosolic free [Mg^2+^]. Therefore, we used patients with McArdle's and Tarui's disease as experimental models to study the pattern of [Mg^2+^] during exercise and recovery in the absence of intracellular acidification to understand to what extent homeostasis of intracellular free Mg^2+ ^is linked to pH. Due to the rare nature of these disorders we were able to enrol just two patients for both diseases and therefore we had to deal with a small sample size. The results show that the increase in cytosolic [Mg^2+^] occurring in skeletal muscle during exercise is actually the consequence of the increase of H^+ ^concentration and not of other mechanisms related to muscle contraction. In addition, we found that both PFK patients showed a reduction of [Mg^2+^] during exercise concomitant with the PME increase. The decrease of [Mg^2+^] also persisted during recovery in PFK I patient who displayed a slower PME recovery. The PME peak in the ^31^P MRS spectra corresponds to the phosphorylated monosaccharide intermediates of glycogenolysis. Therefore, due to the deficit of the phosphofructokinase activity in Tarui's disease, the PME accumulation shown by these patients is likely due to the increase of fructose- 6-phosphate, which represents an additional binding site for cytosolic Mg^2+^. As a consequence, we interpret the decrease of [Mg^2+^] concomitant with the PME increase as due to the binding of Mg^2+ ^to fructose-6-phosphate. A previous study (6) reported that the abnormal PME accumulation of PFK patients during exercise was accompanied by a subnormal Pi accumulation. This finding was interpreted as a result of the incorporation of free Pi into phosphorylated glycolytic intermediates. However, both our patients did not show any Pi trap into PME, since we found that the sum of PCr and Pi was constant for the whole exercise duration, while the total phosphates signal increased proportionally to PME increase. Therefore, the [Mg^2+^] decrease found in PFK patients cannot be ascribed to a diminished Pi build-up.

Moreover, PFK II patient displayed a larger decrease of [Mg^2+^] from rest to end-exercise compared to PFK I patient. Accidentally, PFK II patient also had a smaller PCr breakdown, nevertheless, this cannot be the cause of the larger decrease of [Mg^2+^], as a lower [PCr] corresponds to lower calculated [Mg^2+^] (3).

## Conclusion

Our results show that:

i) free [Mg^2+^] is strongly linked to pH in skeletal muscle homeostasis as previously suggested by a study in healthy volunteers [3], and by computer simulation on a chemical model mimicking muscle cell cytosol [13];

ii) the decrease of free [Mg^2+^] during exercise in both PFK patients suggests that [Mg^2+^] is influenced by the accumulation of fructose- 6-phosphate, an additional binding site for cytosolic Mg^2+^, as shown by the accumulation of the phosphomonoesters peak in the ^31^P MRS spectra of these patients.

iii) ^31^P MRS is a suitable tool for the *in vivo *assessment of free cytosolic [Mg^2+^] in human skeletal muscle during rest, exercise and recovery;

## Authors' contributions

EM participated in the study design, performed the post-processing and statistical analysis, RL and CT participated in the study design and coordinated the data collection, AM participated in the study design, BB participated in the coordination of the study, SI participated in the study design, coordinated the study, and drafted the manuscript. All authors read and approved the final manuscript.
